# Mass cytometric analysis unveils a disease-specific immune cell network in the bone marrow in acquired aplastic anemia

**DOI:** 10.3389/fimmu.2023.1274116

**Published:** 2023-11-29

**Authors:** Emma S. Pool, Yvonne Kooy-Winkelaar, Vincent van Unen, J.H. Frederik Falkenburg, Frits Koning, Mirjam H. M. Heemskerk, Jennifer M-L. Tjon

**Affiliations:** ^1^ Department of Hematology, Leiden University Medical Center, Leiden, Netherlands; ^2^ Department of Immunology, Leiden University Medical Center, Leiden, Netherlands; ^3^ Institute for Immunity, Transplantation, and Infection, Stanford University School of Medicine, Stanford, CA, United States

**Keywords:** aplastic anemia, bone marrow failure, mass cytometry, immunophenotyping, bone marrow, immune cell network

## Abstract

Idiopathic acquired aplastic anemia (AA) is considered an immune-mediated syndrome of bone marrow failure since approximately 70% of patients respond to immunosuppressive therapy (IST) consisting of a course of anti-thymocyte globulin (ATG) followed by long-term use of ciclosporin. However, the immune response that underlies the pathogenesis of AA remains poorly understood. In this study, we applied high-dimensional mass cytometry on bone marrow aspirates of AA patients pre-ATG, AA patients post-ATG and healthy donors to decipher which immune cells may be implicated in the pathogenesis of AA. We show that the bone marrow of AA patients features an immune cell composition distinct from healthy donors, with significant differences in the myeloid, B-cell, CD4^+^ and CD8^+^ T-cells lineages. Specifically, we discovered that AA pre-ATG is characterized by a disease-specific immune cell network with high frequencies of CD16^+^ myeloid cells, CCR6^++^ B-cells, Th17-like CCR6^+^ memory CD4^+^ T-cells, CD45RA^+^CCR7^+^CD38^+^ CD8^+^ T-cells and KLRG1^+^ terminally differentiated effector memory (EMRA) CD8^+^ T-cells, compatible with a state of chronic inflammation. Successful treatment with IST strongly reduced the levels of CD16^+^ myeloid cells and showed a trend toward normalization of the frequencies of CCR6^++^ B-cells, CCR6^+^ memory CD4^+^ T-cells and KLRG1^+^EMRA CD8^+^ T-cells. Altogether, our study provides a unique overview of the immune landscape in bone marrow in AA at a single-cell level and proposes CCR6 as a potential new therapeutic target in AA.

## 1 Introduction

Idiopathic acquired aplastic anemia (AA) is characterized by bone marrow (BM) hypocellularity due to a profound decrease in hematopoiesis resulting in pancytopenia (1). Symptoms of AA relate to the severity of pancytopenia and severe cases of AA are potentially fatal, mainly due to bleeding and infectious complications.

Two effective treatments can result in recovery of hematopoiesis: allogeneic hematopoietic stem cell transplantation (allo-HSCT) and immunosuppressive therapy (IST) (1–3). First-line allo-HSCT leads to rapid recovery of hematopoiesis but has a risk of graft versus host disease and transplant-related mortality and is therefore only recommended for patients under the age of 40 with an HLA-identical sibling donor (4). Accordingly, IST is the preferred first-line treatment in most AA patients.

Standard IST for AA combines ciclosporin and anti-thymocyte globulin (ATG), a polyclonal IgG antibody preparation that is manufactured by immunizing animals with human thymocytes. In the setting of AA both rabbit-derived ATG (rATG) and horse-derived ATG (hATG) have been applied. However, hATG is preferred since hematological response to hATG is superior to rATG (5, 6). In patients responsive to IST, peripheral blood (PB) counts gradually recover within several months. Therefore, international guidelines recommend response evaluation 3-6 months after start of IST. Consequently, non-responding patients are identified after months of prolonged cytopenia and remain at risk for bleeding and infectious complications. One third of patients fail to recover sufficiently after IST (6). At present, it is not possible to predict at diagnosis which patients will respond to IST.

Better understanding of the pathogenesis of AA could aid in timely identifying patients that are less likely to respond to IST. BM recovery after IST strongly implies an immune-mediated pathogenesis in AA. Because ATG results in lymphocyte depletion and effector T-cells are expanded in AA (7), a widely accepted view is that hematopoietic stem and progenitor cells (HSPCs) in the BM are targeted by autoreactive T-cells. However, if AA would only be mediated by T-cells, it is surprising that hATG is superior to rATG, since hATG is less potent at depleting lymphocytes (5, 6). Therefore, we include the possibility that the therapeutic effect of hATG goes beyond T-cell depletion and involves an effect on other immune cell populations as well.

Previous research extensively investigated immune cells in PB in AA and suggested a role for T-cell (8, 9) and B-cell (10) subpopulations in its pathogenesis. However, a detailed overview of the immune landscape at the site of HSPC destruction, the BM, is lacking. The few studies that reported T-cell abnormalities in the BM could not provide a full overview of the immune cell composition due to limitations in the size of flow cytometry antibody panels. In recent years, high-dimensional discovery tools like single-cell RNA sequencing (scRNA-seq) or mass cytometry offered new possibilities to study the immune system in tissues (11). Mass cytometry, in contrast to scRNA-seq, quantifies protein expression levels on single-cells and therefore allows for the exploration of both common and rarer immune subpopulations. In the present study, we applied mass cytometry on BM aspirates of AA patients. This enabled us to reveal a considerable shift in the immune cell composition in BM from AA patients and to identify a disease-specific immune cell network. Our study contributes to our understanding of AA as an immune-mediated form of BM failure and shows that not only T cells, but also myeloid cells and B-cells are likely involved in its pathogenesis.

## 2 Methods

### 2.1 Patients and samples

BM aspirates and PB samples were collected from 7 adult AA patients at diagnosis (pre-ATG; AA^PRE^) and 7 age- and sex-matched healthy donors (HDs). In addition, BM aspirates from 3 of 7 AA patients were collected six months after start of first-line IST (post-ATG; AA^POST^), which consisted of 4 days 40mg/kg ATGAM^®^ (hATG; Pfizer) and 5mg/kg/day ciclosporin for at least six months. BM and PB collection was performed after given informed consent in accordance with the Declaration of Helsinki. Clinical characteristics of patients and HDs are outlined in **Table 1**. All patients were treatment-naïve at diagnosis, had an indication for treatment, did not have an additional underlying disease and were diagnosed and treated according to Dutch guidelines (3). Patients under the age of 40 were screened for Fanconi anemia to rule out a congenital form of BM failure. 1 patient was classified as non-severe AA (NSAA), 2 patients as severe AA (SAA) and 4 patients as very severe AA (VSAA), defined as described in international guidelines (2). Complete response to IST was defined as complete normalization of PB counts. Partial response was defined as transfusion independence and a neutrophil count of at least 0.5·10^9^ neutrophils/L. This study was approved by the Medical Ethical Committee of the Leiden University Medical Center (protocol number B20.037).

**Table 1:** Characteristics of patients and response to IST.

Characteristic	AA^PRE^	AA^POST^	HD
Patients (n)	7	3	7
Age (median, range in years)	31 (23-42)	28 (27-40)	31 (24-44)
Sex (n, M:F)	4:3	2:1	4:3
Severity of disease (n)			
Non-severe AA	1	0	NA
Severe AA	2	1	NA
Very severe AA	4	2	NA

AA indicates aplastic anemia; F, female; IST, immunosuppressive therapy; M, male; NA, not applicable.

### 2.2 Mass cytometry antibody staining, data acquisition and data analysis

Bone marrow mononuclear cells (BMMCs) or PB mononuclear cells (PBMCs) were isolated from BM aspirates or PB, stained with a panel of 39 validated (12–14) metal-isotope tagged antibodies and measured as described in **Supplemental Methods**. For each acquired mass cytometry data file, single, live CD45^+^ cells were gated in FlowJo version 10.7.1 (BD Biosciences) as shown in **Supplementary Figure 1**. A median of 0.5·10^6^ (range 0.4-0.9·10^6^) cells was gated for each AA^PRE^ sample. For every HD or AA^POST^ sample, a median of 0.5·10^6^ (range 0.3-0.9·10^6^) or 0.3·10^6^ (range 0.3-0.6·10^6^) cells was gated respectively.

The single, live CD45^+^ cells were studied using two analyses. In the first analysis, BMMC samples from all AA^PRE^ and HDs were studied. 7.7·10^6^ single, live CD45^+^ cells were sample-tagged, hyperbolic ArcSinh transformed with a cofactor of 5 and imported in Cytosplore (15) for dimensionality reduction. A five-level hierarchical stochastic neighbor embedding (HSNE) analysis was performed with default perplexity and iterations (30 and 1000, respectively; see **Supplementary Figure 2** for outline of HSNE analysis pipeline). Three of 39 markers in the mass cytometry panel (HLA-DR, CD5 and KLRG1) were not used in the HSNE clustering analysis due to small variations in marker distribution between experiments (data not shown). Major immune lineages were identified at the overview level of the HSNE analysis and were defined as: CD4^+^CD3^+^CD7^+^ CD4^+^ T-cells, CD8a/b^+^CD3^+^CD7^+^ CD8^+^ T-cells, CD7^-^CD20/IgM^+^ B-cells, CD3^+^CD7^+^TCRgd^+^ non-conventional T-cells (NCTs), CD3^-^CD7^+^ innate lymphoid cells (ILCs) including NK-cells and Lin^-^cKit^+^ HSPCs. All remaining cells were considered myeloid cells. Subsequently, each major immune lineage was selected for detailed analysis at the data level of the HSNE analysis using t-distributed stochastic neighbor embedding (tSNE) analyses with up to 0.5·10^6^ landmarks. Clusters with distinct surface marker expression were identified within each major immune lineage by Gaussian mean shift (GMS) clustering at the data level. Each cluster consisted of at least 1000 cells (**Supplementary Figure 2**).

In the second analysis, paired BMMC samples from 3 AA^PRE^ and AA^POST^ were analyzed using a comparable approach, for detailed description see **Supplemental Methods**.

After clustering in both analyses, .fcs files of major immune lineages and clusters were exported from Cytosplore and loaded into R version 4.0.5 for data visualization and analysis. Cytofast (16) was used to generate heatmaps and to compare lineage or cluster frequencies between groups. Principal component analyses (PCA) and a Spearman’s rank correlation analysis were performed using the packages factoextra version 1.0.7 (17) and corrplot version 0.92 (18), respectively.

### 2.3 Statistical analysis

Mann-Whitney *U* tests performed in R version 4.0.5 were used to compare immune cell frequencies between two groups. *P*-values ≤0.05 were considered statistically significant. Since this was an exploratory study, no adjustments for multiple testing were applied. *P ≤ 0.05; **P ≤ 0.01; ***P ≤ 0.001.

## 3 Results

### 3.1 The immune cell composition in bone marrow of AA patients pre-ATG is distinct from healthy donors

**Figure 1:**
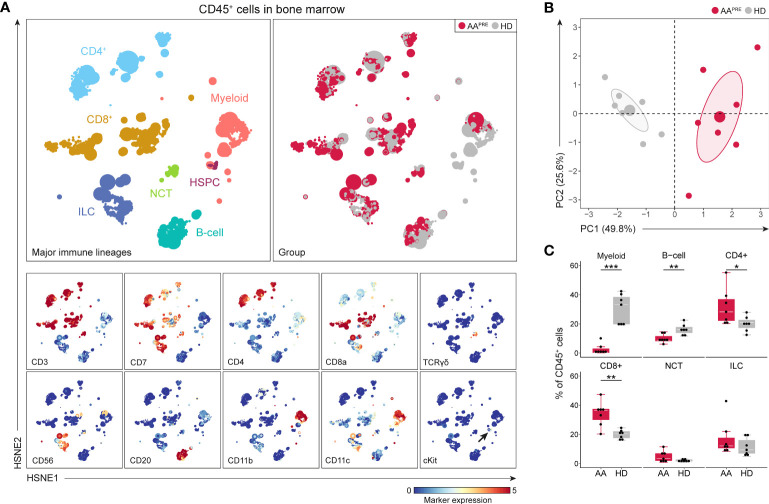
Major immune lineages in BM in AA pre-ATG and HD. Colors indicate major immune lineages (HSPCs, myeloid cells, B-cells, CD4^+^ T-cells, CD8^+^ T-cells, NCT-cells and ILCs), groups (AA^PRE^ in red and HDs in gray) or median ArcSinh-transformed marker expression values. **(A)** Major immune lineages in BM of 7 AA^PRE^ and 7 HDs, visualized at the overview level of a five-level HSNE analysis. 7.8·10^3^ HSNE landmarks represented all 7.7·10^6^ CD45^+^ cells in the analysis. The size of each landmark reflects the number of cells represented by the landmark. cKit^+^ cells are indicated by an arrow. Major immune lineages were defined as following: CD4^+^CD3^+^CD7^+^ CD4^+^ T-cells, CD8a/b^+^CD3^+^CD7^+^ CD8^+^ T-cells, CD7^-^CD20/IgM^+^ B-cells, CD3^+^CD7^+^TCRgd^+^ non-conventional T-cells (NCTs), CD3^-^CD7^+^ innate lymphoid cells (ILCs) including NK-cells and Lin^-^cKit^+^ HSPCs. All remaining cells were considered myeloid cells. **(B)** Principal component analysis of the 7 AA^PRE^ and 7 HD BM samples. Each larger dot represents the group mean. Each smaller dot represents a sample. **(C)** Major immune lineage frequencies in AA^PRE^ and HDs, shown as a percentage of all CD45^+^ cells in BM. Each black dot represents a sample. Box plots present the median with the interquartile range (IQR), error bars present 1.5*IQR. *P ≤ 0.05; **P ≤ 0.01; ***P ≤ 0.001.

First, we studied the immune cell composition in the BM of the 7 AA^PRE^ and 7 age- and sex-matched HDs. A five-level HSNE analysis was performed to visualize all 7.7·10^6^ CD45^+^ cells (median 0.5·10^6^ CD45^+^ cells per sample). At the overview level of the HSNE analysis, all major immune lineages and HSPCs could be identified by clustering based on lineage markers and cKit (**Figure 1A**). All AA^PRE^ and HD samples were homogeneously distributed per group over the HSNE analysis (**Supplementary Figure 3**). Visualization of the AA^PRE^ and HDs in the overview level already revealed clear differences in the immune cell composition.

An unsupervised PCA confirmed a clear separation in the immune cell composition of AA^PRE^ and HDs (**Figure 1B**). Next, the immune cell composition was studied in further detail at the major immune lineage level. Since we aimed to uncover how the immune cell composition differs in BM from AA^PRE^ and HDs and total cell counts are decreased in the BM in AA, we studied relative cell frequencies instead of absolute cell counts. Major immune lineage frequencies of CD45^+^ cells were determined (**Figure 1C**). HSPCs could not be distinguished enough at this level of the HSNE analysis and were therefore merged with myeloid cells. In agreement with disease characteristics, myeloid cells were scarce in AA^PRE^ compared to HDs (median 0.6% *versus* 33% of CD45^+^ cells). B-cells were also reduced in AA^PRE^ compared to HDs (median 9% *versus* 16% of CD45^+^ cells). In contrast, CD4^+^ and CD8^+^ T-cells were increased in AA^PRE^ compared to HDs, which could be due to the relative decrease in myeloid and B-cells. Finally, non-conventional T-cell (NCT) and innate lymphoid cell (ILC) frequencies did not significantly differ between AA^PRE^ and HDs.

### 3.2 Stem cell depletion and high frequencies of CD16^+^ myeloid cells in AA pre-ATG

**Figure 2:**
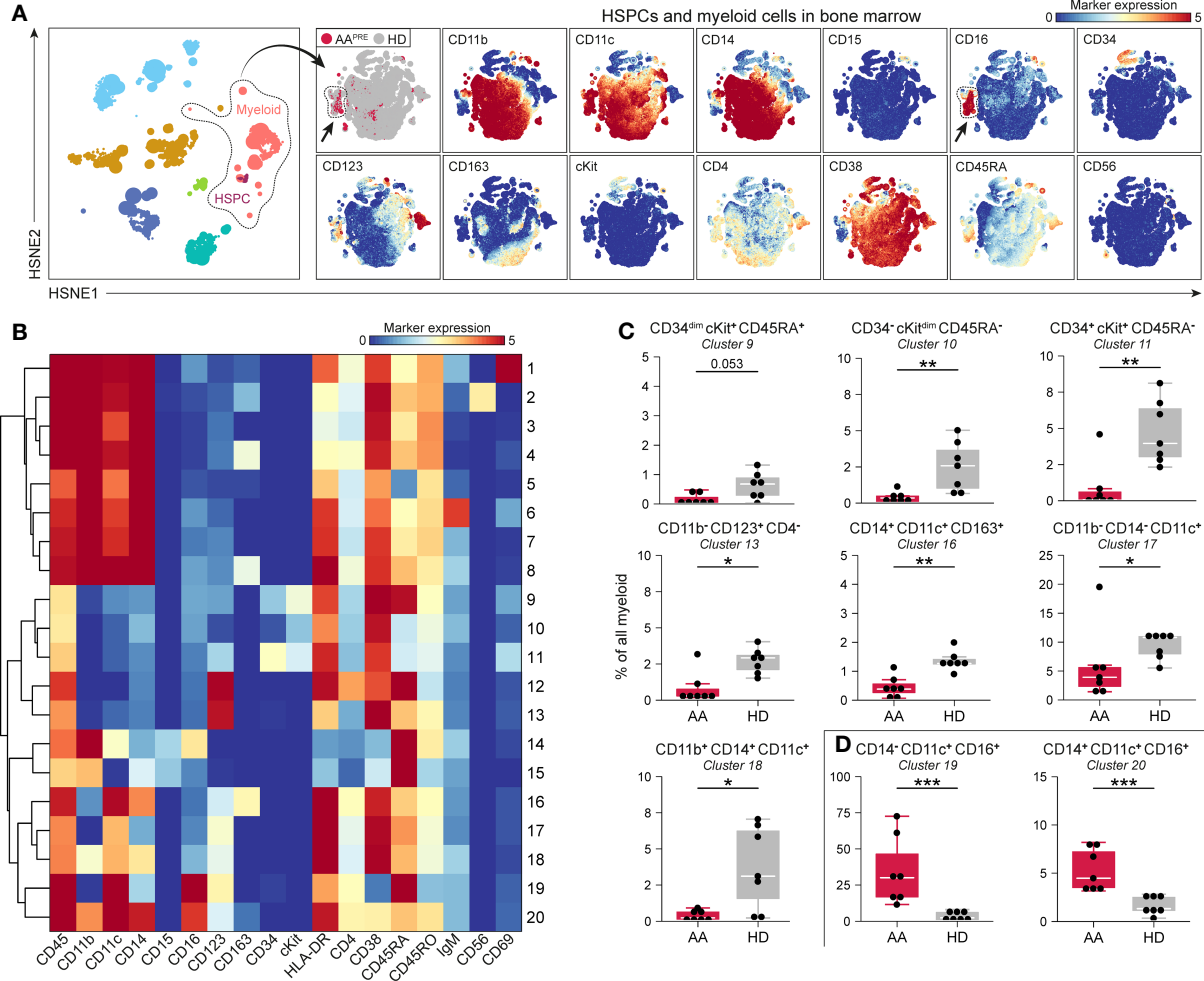
Myeloid cell compartment including HSPCs in BM in AA pre-ATG and HD. Colors indicate major immune lineages (HSPCs, myeloid cells, B-cells, CD4+ T-cells, CD8+ T-cells, NCT-cells and ILCs), groups (AA^PRE^ in red or HDs in gray) or median ArcSinh-transformed marker expression values. **(A)** HSPCs and myeloid cells isolated from BM of 7 AA^PRE^ and 7 HDs, visualized at level four of a five-level HSNE analysis. 2.4·10^5^ landmarks represent 1.3·10^6^ cells. The size of each landmark reflects the number of cells represented by the landmark. Arrows indicate CD16^+^ myeloid cells. **(B)** Heatmap presenting median ArcSinh-transformed marker expression values for the 20 clusters identified within the HSPC and myeloid cell compartment. The relationships of similarity between clusters are depicted by the dendrogram. **(C)** HSPC and myeloid cell clusters reduced in AA^PRE^. **(D)** HSPC and myeloid cell clusters increased in AA^PRE^. Cluster frequencies are presented as a percentage of all HSPCs and myeloid cells in BM from each respective patient or HD. Each black dot represents a sample. Box plots present the median with the interquartile range (IQR), error bars present 1.5*IQR. Only clusters that significantly differed in frequency between AA^PRE^ and HDs are shown. *P ≤ 0.05; **P ≤ 0.01; ***P ≤ 0.001.

Since myeloid, B-cell, CD4^+^ T-cell and CD8^+^ T-cell frequencies were significantly different in BM from AA^PRE^ compared to HDs, we studied each of these lineages at the immune subpopulation level. First, the myeloid cells including HSPCs were selected and analyzed at the four successive levels of the HSNE analysis (**Figure 2**, **Supplementary Figure 2**). In this manner, multiple HSPC and myeloid cell populations could be distinguished based on the expression of defining cell surface markers (**Figure 2A**, right panels). Twenty phenotypically distinct clusters were identified (**Figure 2B**), 8 of which were significantly different in AA^PRE^ compared to HDs, 1 had a p-value of 0.053 and 11 clusters did not significantly differ between both groups. Analysis of the 8 significant clusters and cluster 9 showed a strong reduction in all cKit^+/dim^ clusters (#9-11) in AA^PRE^, confirming HSPCs depletion in AA^PRE^ (**Figure 2C**). Furthermore, we observed a decrease of myeloid cells compatible with CD123^+^ plasmacytoid dendritic cells (#13), CD163^+^ macrophages (#16), CD11c^+^ conventional dendritic cells (#17) and CD14^+^CD16^-^ classical monocytes (#18) in AA^PRE^. The most striking observation was a substantial increase in CD16^+^ myeloid cells in AA^PRE^, which lacked expression of the neutrophil marker CD15 and represented a median of 41% (range 16-83%) of all myeloid cells (**Supplementary Figures 4A, B** #14;19-20 combined; arrows in **Figure 2A**). Two CD16^+^ myeloid cell clusters (#19-20) resembling CD14^-^CD16^+^ non-classical and CD14^+^CD16^+^ intermediate monocytes were significantly increased in AA^PRE^ (**Figure 2D**). In contrast, CD16^+^ myeloid cells only comprised a median of 5% (range 2-19%) of all myeloid cells in HDs (**Supplementary Figures 4A, B**).

### 3.3 CCR6^++^ B-cells predominate in AA pre-ATG

**Figure 3:**
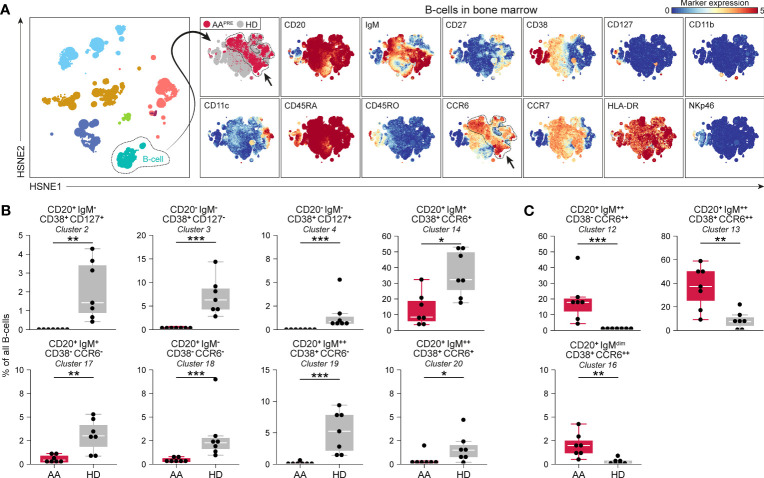
B-cell compartment in BM in AA pre-ATG and HD. Colors indicate major immune lineages (HSPCs, myeloid cells, B-cells, CD4^+^ T-cells, CD8^+^ T-cells, NCT-cells and ILCs), groups (AA^PRE^ in red or HDs in gray) or median ArcSinh-transformed marker expression values. **(A)** B-cells isolated from BM of 7 AA^PRE^ and 7 HDs, visualized at level four of a five-level HSNE analysis. The HSNE depicts 1.6·10^5^ landmarks for 0.9·10^6^ B-cells. The size of each landmark reflects the number of cells represented by the landmark. Arrows indicate CCR6^++^ B-cells. **(B)** B-cell clusters reduced in AA^PRE^. **(C)** B-cell clusters increased in AA^PRE^. Cluster frequencies are presented as a percentage of all B-cells in BM from each respective patient or HD. Each black dot represents a sample. Box plots present the median with the interquartile range (IQR), error bars present 1.5*IQR. Only clusters that significantly differed in frequency between AA^PRE^ and HDs are shown. *P ≤ 0.05; **P ≤ 0.01; ***P ≤ 0.001.

Detailed analyses of all B-cells revealed phenotypical heterogeneity within the B-cell compartment (**Figure 3A**). 20 B-cell clusters were identified (**Supplementary Figure 4C**). CD20^+^IgM^-^CD127^+^ B-cells (#2) and CD20^-^IgM^-^ B-cells expressing variable levels of CD127 (#3-4), compatible with a group of early B-cells (19, 20), were markedly reduced in AA^PRE^ compared to HDs (**Figure 3B**). We also observed a decrease in B-cells with absent or low expression of the chemokine receptor CCR6 (**Figure 3B**, #14;17-20). Strikingly, B-cells expressing high levels of CCR6 were strongly increased in AA^PRE^ compared to HDs (**Figure 3C**, #12-13;16). Combined, CCR6^++^ B-cells constituted a median of 64% (range 38-86%) of all B-cells in AA^PRE^ and only a median of 10% (range 1-26%) of all B-cells in HDs (**Supplementary Figures 4C, D**, #5;9;12-13;16 combined). Clusters 12 and 16 co-expressed CD27 in line with previous descriptions of memory B-cells (21–23) (**Supplementary Figure 4C**). In contrast, cluster 13 lacked expression of CD27, compatible with a population of immature or mature B-cells (24). In addition, the CCR6^++^ B-cells expressed different levels of IgM, indicating that these cells were in multiple stages of development.

### 3.4 Th17-like CCR6^+^CD4^+^ T-cells are increased in AA pre-ATG

**Figure 4:**
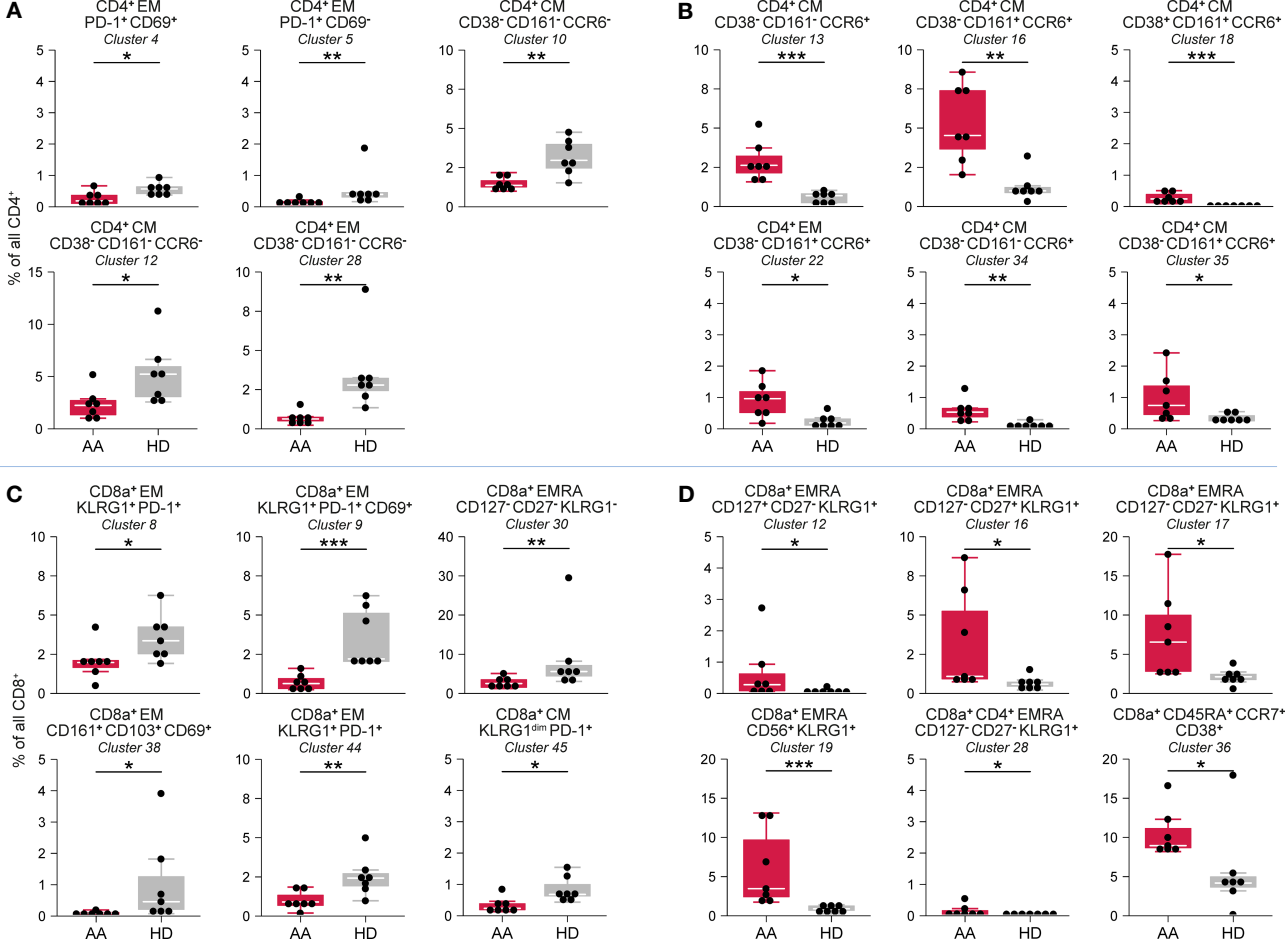
CD4^+^ T-cell and CD8^+^ T-cell compartment in BM in AA pre-ATG and HD. Colors indicate groups (AA^PRE^ in red or HDs in gray). **(A)** CD4^+^ T-cell clusters reduced in AA^PRE^. **(B)** CD4^+^ T-cell clusters increased in AA^PRE^. **(C)** CD8^+^ T-cell clusters reduced in AA^PRE^. **(D)** CD8^+^ T-cell clusters increased in AA^PRE^. Cluster frequencies are presented as a percentage of all CD4^+^ T-cells or CD8^+^ T-cells in BM from each respective patient or HD. Each black dot represents a sample. Box plots present the median with the interquartile range (IQR), error bars present 1.5*IQR. Only clusters that significantly differed in frequency between AA^PRE^ and HDs are shown. *P ≤ 0.05; **P ≤ 0.01; ***P ≤ 0.001.

Hierarchical clustering of all CD4^+^ T-cells classified 35 phenotypically distinct CD4^+^ T-cell clusters in different stages of development (**Supplementary Figures 4E–G**). No significant differences were observed in naïve (CD45RA^+^CCR7^+^) CD4^+^ T-cell clusters. In contrast, multiple central memory (CM; CD45RO^+^CCR7^+^) and effector memory (EM; CD45RO^+^CCR7^-^) CD4^+^ T-cell clusters were significantly reduced in AA^PRE^ (**Figure 4A**). The most remarkable difference was an increase in CM and EM CD4^+^ T-cells with a Th17-like phenotype based on the co-expression of CCR6 and/or CD161 (25), suggestive of Th17-skewing in AA^PRE^. Six CCR6^+^CD4^+^ T-cell clusters were significantly increased in AA^PRE^ compared to HDs (**Figure 4B**). Together, the CCR6^+^CD4^+^ T-cells represented a median of 11% (range 5-18%) of CD4^+^ T-cells in AA^PRE^, compared to a median of 2% (range 2-6%) of CD4^+^ T-cells in HDs (**Supplementary Figures 4F, G**, #13;16;18;20;22;34-35 combined).

### 3.5 Increase of KLRG1^+^ terminally differentiated effector memory CD8^+^ T-cells in AA pre-ATG

Hierarchical clustering of all CD8^+^ T-cells identified 45 phenotypically distinct CD8^+^ T-cell clusters (**Supplementary Figures 4H–J**). CM and EM CD8^+^ T-cells were predominantly reduced in AA^PRE^ (**Figure 4C**, **Supplementary Figures 4I, J**). A cluster of CD8^+^ T-cells (#36) resembling a population of naïve but activated (CD38^+^) T-cells was significantly increased in AA^PRE^ (**Figure 4D**). The most striking observation within the CD8^+^ T-cell compartment in AA^PRE^ was an increase of EMRA CD8^+^ T-cells positive for KLRG1 resembling highly differentiated cytotoxic T-cells (26). These KLRG1^+^EMRA CD8^+^ T-cells constituted a median of 29% (range 15-45%) of all CD8^+^ T-cells in AA^PRE^, but only a median of 9% (range 7-33%) of all CD8^+^ T-cells in the HDs (#10-22;24-26;28-29 combined; **Supplementary Figures 4I–J**). Five KLRG1^+^EMRA CD8^+^ T-cell clusters (#12;16-17;19;28) were significantly elevated in AA^PRE^ compared to HDs (**Figure 4D**).

### 3.6 A disease-specific immune cell network characterizes AA pre-ATG

**Figure 5:**
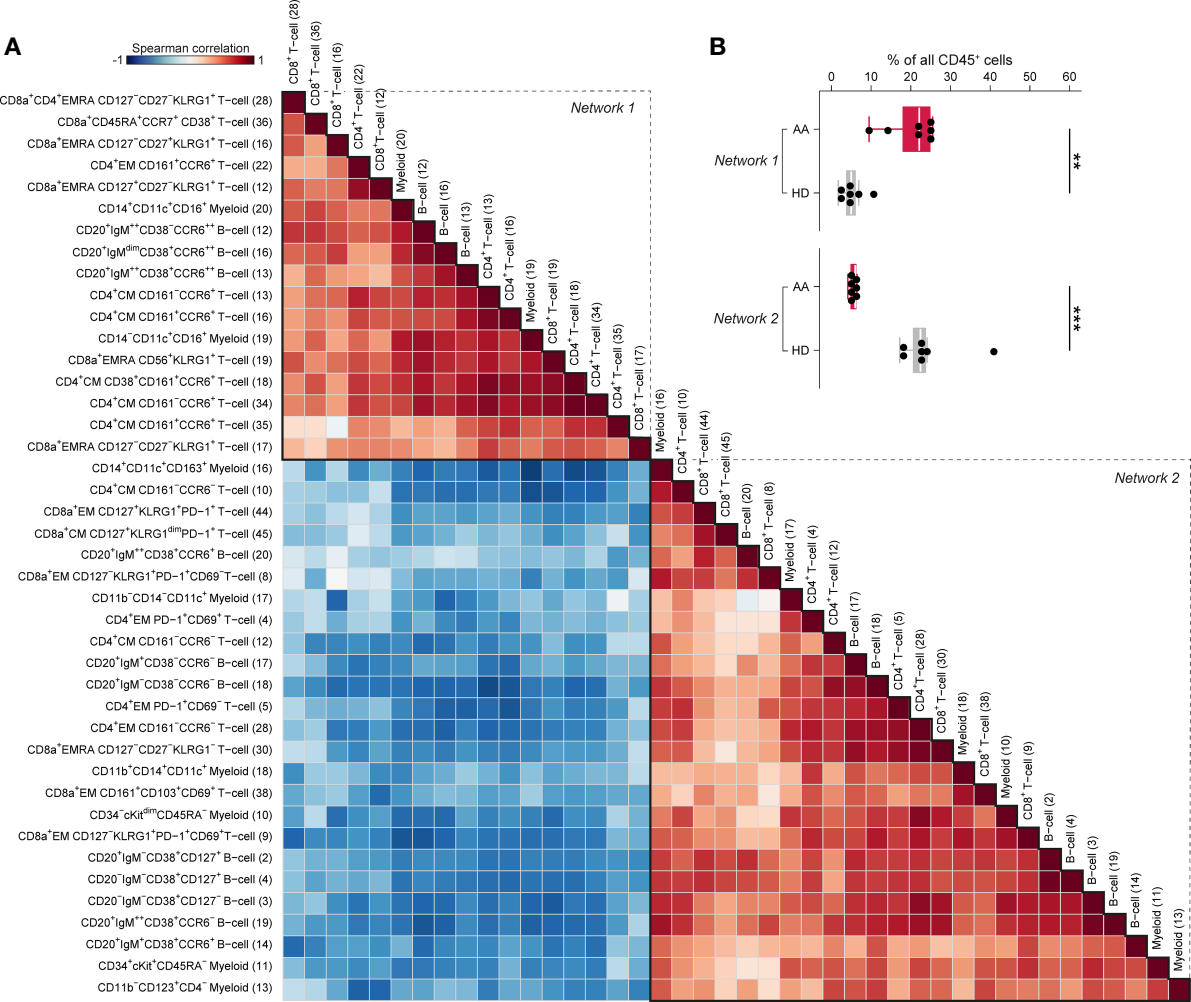
Spearman’s rank correlation analysis of differentially present myeloid, B-cell and T-cell clusters in BM in AA pre-IST and HD. **(A)** Spearman’s rank correlation analysis based on the frequencies of all differentially present cell clusters in AA^PRE^ compared to HDs. All AA^PRE^ (n=7) and HDs (n=7) samples were included in the analysis. The strength of the correlations is presented in color (blue to red). The phenotype of each cell cluster is presented left of the correlation matrix. The number of each cell cluster is shown between brackets and corresponds to the cell cluster numbers used in **Figures 2**, **4** and **Supplementary Figure 4**. **(B)** Total frequency of all cell clusters in networks 1 or 2, expressed as a percentage of all CD45^+^ cells in BM from each respective patient or HD. Colors indicate groups (AA^PRE^ in red or HDs in gray). Each black dot represents a sample. Box plots present the median with the interquartile range (IQR), error bars present 1.5*IQR. *P ≤ 0.05; **P ≤ 0.01; ***P ≤ 0.001.

To capture the relationships across the immune cell clusters that characterize AA^PRE^, we performed a Spearman’s rank correlation analysis on the myeloid, B-cell and T-cell clusters with significant differential presence between AA^PRE^ and HDs. A matrix of Spearman’s correlation coefficients visualized two large networks of immune cells (**Figure 5A**). In line with the analysis of the major immune lineages above, network 1 showed a correlation between CD16^+^ myeloid cells, CCR6^++^ B-cells, CCR6^+^ memory CD4^+^ T-cells, CD45RA^+^CCR7^+^CD38^+^ CD8^+^ T-cells and KLRG1^+^EMRA CD8^+^ T-cells, and was significantly more abundant in AA^PRE^ compared to HDs (median 22% *versus* 5% of CD45^+^ cells, **Figure 5B**). In contrast, network 2 was significantly reduced in AA^PRE^ compared to HDs (median 6% *versus* 23% of CD45^+^ cells). Analysis of paired PB and BM samples from all 7 AA^PRE^ demonstrated that all subpopulations of network 1 are detectable in PB (**Supplementary Figure 5**).

### 3.7 IST normalizes the immune cell composition in patients with adequate hematological improvement six months post-ATG

Since IST can result in recovery of hematopoiesis, we investigated the effect of IST on the immune cell composition in BM of 3 AA patients pre- and six months post-ATG. All patients still received ciclosporin and were transfusion-independent six months post-ATG. All patients had improvement of PB counts and eventually responded to IST, however, only one patient (AA3) showed complete response six months post-ATG (8.3 mmol hemoglobulin/L, 149·10^9^ platelets/L and 1.8·10^9^ neutrophils/L). One patient (AA2) had major partial response six months post-ATG (5.8 mmol hemoglobulin/L, 87·10^9^ platelets/L and 1.9·10^9^ neutrophils/L), while the third patient (AA1) showed minor partial response six months post-ATG (5.4 mmol hemoglobulin/L, 18·10^9^ platelets/L and 0.3·10^9^ neutrophils/L).

**Figure 6:**
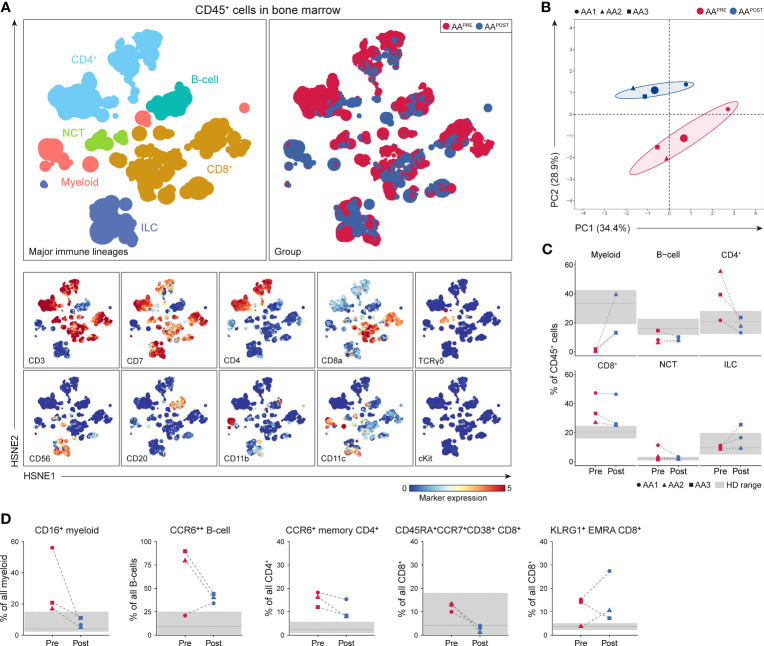
Identification and frequencies of major immune lineages in BM in AA pre- and post-ATG. Colors indicate major immune lineages (myeloid cells, B-cells, CD4^+^ T-cells, CD8^+^ T-cells, NCT-cells and ILCs), groups (AA^PRE^ in red and AA^POST^ in blue) or median ArcSinh-transformed marker expression values. **(A)** Major immune lineages visualized at the overview level of a four-level HSNE analysis with 3.0·10^6^ CD45^+^ cells isolated from paired BM samples collected from 3 AA patients before and six months post-ATG. 1.7·10^4^ landmarks represented all 3.0·10^6^ CD45^+^ cells in the analysis. The size of each landmark reflects the number of cells represented by the landmark. **(B)** Principal component analysis of the 3 AA^PRE^ and 3 AA^POST^ samples. Each larger dot represents the group mean. Each smaller dot represents a sample. **(C)** Major immune lineage frequencies in the AA^PRE^ and AA^POST^ samples, shown as a percentage of all CD45^+^ cells in BM from each respective patient or HD. **(D)** Total pre- and post-ATG frequencies of the CD16^+^ myeloid cell, CCR6^++^ B-cell, CCR6^+^ CM or EM CD4^+^ T-cell, CD45RA^+^CCR7^+^CD38^+^ CD8^+^ T-cell and KLRG1^+^ EMRA CD8^+^ T-cell clusters representing the immune cell network that was significantly increased in AA^PRE^ compared to HDs. Pre- and post-ATG samples of every AA patient are connected by a dotted line. Minimum and maximum ranges of shaded regions depict the minimum and maximum lineage or subpopulation frequencies in the 7 HDs. Median frequencies in HDs are depicted by a solid gray line. Respective patients are represented by different shapes (AA1 closed circle, AA2 triangle, AA3 square). No *P*-values are shown because of the small group size.

A total of 3.0·10^6^ CD45^+^ cells (median 0.5·10^6^ or 0.3·10^6^ cells per AA^PRE^ or AA^POST^, respectively) were analyzed using a four-level HSNE analysis. At the overview level, the global immune cell composition in AA^PRE^ and AA^POST^ was visualized and all major immune lineages were identified (**Figure 6A**). HSPCs were not observed, suggesting that the frequency of HSPCs was too low to be detected. Unsupervised PCA presented a clear separation of AA^PRE^ and AA^POST^ (**Figure 6B**), indicative of a considerable shift in the immune cell composition in the BM six months post-ATG. Analyses at the major immune lineage level demonstrated that myeloid cells were strongly increased or normalized in all AA^POST^ (**Figure 6C**). In addition, CD4^+^ T-cell were normalized, while B-cells were still below normal levels in all AA^POST^. CD8^+^ T-cells remained high in the patient (AA1) with the lowest blood counts six months post-ATG (**Figure 6C**).

To analyze the effect of IST on the disease-specific immune cell network identified in AA^PRE^ (network 1), we studied the presence of its corresponding immune cell subtypes in AA^POST^. Cluster frequencies were compared to frequencies observed in the HDs. Strikingly, this revealed that CD16^+^ myeloid cell frequencies normalized in all AA^POST^ and CD45RA^+^CCR7^+^CD38^+^ CD8^+^ T-cell frequencies decreased in all AA^POST^ (**Figure 6D**). CCR6^++^ B-cell, CCR6^+^ memory CD4^+^ T-cell and KLRG1^+^EMRA CD8^+^ T-cell frequencies showed a trend toward normalization in the two patients (AA2 and AA3) with complete response or major partial response six months post-ATG. In contrast, these clusters were still frequent or increased in the patient (AA1) with minor partial response six months post-ATG (**Figure 6D**).

## 4 Discussion

The present study provides a comprehensive comparison of the immune landscape in BM from AA^PRE^ and HDs with the aim to decipher which immune cells may be implicated in the pathogenesis of AA. We demonstrate that the BM from AA^PRE^ harbors a distinct immune cell composition compared to HDs, with significant differences in the myeloid, B-cell, CD4^+^ T-cell and CD8^+^ T-cell lineages. Detailed analyses at the immune subpopulation level identified a disease-specific immune cell network of CD16^+^ myeloid cells, CCR6^++^ B-cells, Th17-like CCR6^+^CD4^+^ T-cells, CD45RA^+^CCR7^+^CD38^+^ CD8^+^ T-cells and KLRG1^+^EMRA CD8^+^ T-cells that distinguishes AA^PRE^ from HDs. This network showed a trend toward normalization in patients with hematological improvement six months post-ATG, which implies a pathogenic role of the network in AA.

To date, cytotoxic T-cells have been the focus of immunophenotyping studies in AA. The present study shows CD4^+^ and CD8^+^ T-cell derangements in AA^PRE^, consistent with the view that AA is mediated by an autologous T-cell response. The identified disease-specific immune cell network featured a group of KLRG1^+^EMRA CD8^+^ T-cells, which accords with previous reports on autoimmune disorders (27, 28) where KLRG1^+^CD8^+^ T-cells accumulate at inflammatory sites. Although CD57 expression was not evaluated in our study, the KLRG1^+^CD8^+^ T-cells could resemble previous descriptions of CD57^+^CD28^-^ CD8^+^ T-cells, which commonly express KLRG1 (29) and are expanded in PB (30) in AA where their presence seems to correlate with disease activity (31). We assume, therefore, that the identified KLRG1^+^CD8^+^ T-cells have cytotoxic capacity. The target of this immune response remains presently unknown and would be an interesting topic for future studies.

Within the CD4^+^ T-cell compartment, we observed increased frequencies of CCR6^+^ Th17-like cells in the BM of AA^PRE^ compared to HDs. Previous studies postulated a role for Th17 cells in the pathogenesis of AA and identified Th17 cells based on the expression of IL-17 (8, 32) or CD161 (9). Our data are in line with these studies and add CCR6 as a new marker to delineate Th17 cells in AA. Functionally, Th17 cells may contribute to the immune response in AA through their ability to secrete pro-inflammatory cytokines including IL-17 and IL-21 which can trigger macrophage recruitment and activation.

Interestingly, B-cells are not well characterized in AA, even though B-cell involvement is increasingly recognized in autoimmune disorders. We identified high proportions of CCR6^++^ B-cells that have not been described in AA before but are profoundly increased in autoimmune disorders (33). Together with the identification of Th17-like CCR6^+^CD4^+^ T-cells, this presents CCR6 as a potentially important marker in the pathogenesis of AA. CCR6 is a chemokine receptor expressed on various immune cell types and mediates immune cell recruitment to inflammatory sites through CCL20, its sole ligand (34). Increased levels of CCL20 have been described in the BM in AA (35). Hence, it is tempting to speculate that the CCR6-CCL20 axis fuels an immune response in AA, as proposed in other autoimmune disorders (36, 37), by recruiting pro-inflammatory immune cells to the BM.

Autoimmune responses are generally characterized by an expansion of effector T-cells, rather than an accumulation of polyclonal, antigen-inexperienced naïve T-cells. Therefore, it is surprising that our results point to the importance of a cluster of CD45RA^+^CCR7^+^CD38^+^ CD8^+^ T-cells with a naïve phenotype. Since previous work has demonstrated the increased presence of CD95^+^ stem cell-like memory T-cells (T_SCM_) within the naïve CD8^+^ T-cells compartment in AA (38), we consider the possibility that the CD45RA^+^CCR7^+^CD38^+^ CD8^+^ T-cells are CD8^+^ T_SCM_. T_SCM_ are endowed with an enhanced capacity for self-renewal and control immune responses through their ability to generate memory and effector T-cells (39). However, because our panel did not include T_SCM_-specific markers, the precise identity of the CD45RA^+^CCR7^+^CD38^+^ CD8^+^ T-cells remains unclear.

The presence of CD16^+^ myeloid cells in the identified immune cell network is striking because AA is characterized by a profound reduction of the myeloid compartment in PB. Therefore, it would be interesting to determine whether these cells are expanded or are the only myeloid cells left in the BM after immune-mediated destruction in AA. Higher percentages of CD16^+^ macrophages, defined by the expression of CD68 and lower levels of CD14, have been reported in BM in AA, and were suggested to exert BM destructive effects by stimulating effector T-cells through the secretion of TNF-α (40). We characterized the CD16^+^ myeloid cells in further detail and identified two populations that expressed different levels of CD11b and CD14 and did not express the M2-macrophage marker CD163. Based on this expression profile, these CD16^+^ myeloid cells could represent a population of CD14^+^CD16^+^ intermediate and CD14^-^CD16^+^ non-classical monocytes, which can express CD68 (20), produce TNF-α (41) and contribute to autoimmune disorders (42, 43). Regarding the function of these cells, one could speculate whether these cells have a direct role in HSPC destruction, e.g. by cytokine secretion, or that they are recruited to clear dead and dying cells, e.g. by antibody-dependent cell-mediated cytotoxicity, and merely reflect a consequence of the immune response.

Altogether, the disease-specific immune cell network identified in the BM from AA^PRE^ is compatible with a state of chronic inflammation. Although this study did not aim to interrogate the functional role of the network, our post-ATG data could indicate a distinct role of CD16^+^ myeloid cells compared to CCR6^++^ B-cells, Th17-like CCR6^+^CD4^+^ T-cells and KLRG1^+^EMRA CD8^+^ T-cells. ATG-based IST can result in the recovery of hematopoiesis, which implies that IST intervenes with the immune response that underlies the pathogenesis of AA. We demonstrate that CD16^+^ myeloid cells were normalized six months post-ATG, but that CCR6^++^ B-cells, CCR6^+^ Th17-like CD4^+^ T-cells and KLRG1^+^EMRA CD8^+^ T-cells remained elevated in patients with hematological improvement, despite a trend toward normalization. A possible explanation for the fact that only CD16^+^ myeloid cells were strongly reduced six months post-ATG could be that these cells are charged with clearing dead and dying cells in the BM in AA, which would explain the decrease in these cells once the initiating immune response has resolved. However, the decrease in CD16^+^ myeloid cells could also indicate that these cells carry out an effector function in the immune response in AA via cytokine secretion. This would explain the recovery of hematopoiesis after the depletion of CD16^+^ myeloid cells six months post-ATG. As ciclosporin suppresses B-cell (44) and T-cell development (45), we hypothesize that ciclosporin suppresses the CCR6^++^ B-cells, Th17-like CCR6^+^CD4^+^ T-cells and KLRG1^+^EMRA CD8^+^ T-cells that are still elevated six months post-ATG to maintain stable hematopoiesis. The fact that ciclosporin suppression is needed beyond six months could implicate a role of these cells in the initiation of the inflammatory process. Regarding the potential significance of CCR6^++^ B-cells, it is tempting to speculate that hATG-based IST, the preferred first-line treatment in AA, may be more potent at depleting CCR6^++^ B-cells than rATG-based IST and is, therefore, superior in the setting of AA. Based on the potential importance of CCR6^++^ B-cells and CCR6^+^ Th17-like CD4^+^ T-cells, our study proposes CCR6 as a new potential therapeutic target. Blockade of CCR6 and disruption of the CCR6-CCL20 interaction have been shown to reduce severity of several autoimmune disorders (46, 47). Multiple reports describe CCR6 blockade therapies targeting pathogenic Th17 T-cells (46, 48) and show improvement in diseases such as psoriasis (47) through the inhibition of T-cell recruitment to inflammatory sites, illustrating that CCR6 blockade could be effective in treating T-cell mediated autoimmune disorders. Another report proposes a small molecule inhibitor of CCR6 as a potential therapy for autoimmune disorders through its ability to inhibit CCR6-dependent B-cell migration (49). Whether anti-CCR6 treatment may be used to control inflammation and symptoms in the active phase of AA could be subject of further studies.

To investigate the clinical applicability of our work, we analyzed PB for the presence of the disease-specific immune cell network and found that all subpopulations of the immune cell network are detectable in paired PB and BM samples from AA^PRE^ The fact that the disease-specific immune cell network can be measured in PB offers possibilities for its clinical use as a marker of disease activity to evaluate the success of IST and possible disease progression.

In summary, we identified a disease-specific immune cell network in the BM from AA^PRE^. While inflammation-associated immune cells were expected in AA, our approach shows for the first time an association between CD16^+^ myeloid cells, Th17-like cells, CD45RA^+^CCR7^+^CD38^+^ CD8^+^ T-cells and KLRG1^+^EMRA CD8^+^ T-cells and adds CD16^+^ myeloid cells and CCR6^++^ B-cells as a potentially important population in AA. Successful treatment with IST strongly reduced the levels of CD16^+^ myeloid cells and showed a trend toward normalization of the frequencies of CCR6^++^ B-cells, CCR6^+^ memory CD4^+^ T-cells and KLRG1^+^EMRA CD8^+^ T-cells. The identified network has the potential to be used as a marker for disease activity at diagnosis and during treatment of AA. Together, our study sets the stage for further exploration of the functional relevance and cellular spatial interactions of this immune cell network within the bone marrow microenvironment.

## Data availability statement

Mass cytometry data acquired in this study have been deposited to https://flowrepository.org (ID: FR-FCM-Z7YJ).

## Ethics statement

The studies involving humans were approved by Medical Ethics Committee Leiden The Hague Delft. The studies were conducted in accordance with the local legislation and institutional requirements. The participants provided their written informed consent to participate in this study.

## Author contributions

EP: Formal Analysis, Investigation, Visualization, Writing – original draft. YK-W: Investigation, Writing – review & editing. VvU: Investigation, Methodology, Writing – review & editing. JF: Investigation, Writing – review & editing. FK: Investigation, Supervision, Writing – review & editing. MH: Investigation, Supervision, Writing – review & editing. JT: Conceptualization, Funding acquisition, Methodology, Resources, Supervision, Writing – review & editing.
