# Supplementary material for: Mass cytometric analysis unveils a disease-specific immune cell network in the bone marrow in acquired aplastic anemia
Source: Front Immunol. 2023 Nov 29;14:1274116. doi: 10.3389/fimmu.2023.1274116 (PMC10716190; doi:10.3389/fimmu.2023.1274116)
Supplement: Supplementary file 1 [file DataSheet_1.pdf]

Supplementary Material

1 Supplementary material

1.1 Supplementary methods

1.1.1 Mononuclear cell isolation and cryopreservation

Bone marrow mononuclear cells (BMMCs) or PB mononuclear cells (PBMCs) were isolated from fresh BM aspirates or PB using Ficoll separation gradient or CPT Cell Preparation Tubes (BD, cat 362780). Subsequently, cells were cryopreserved in liquid nitrogen until further use.

1.1.2 Mass cytometry antibodies

Mass cytometry antibodies and antibody specifications are listed in Supplementary Table 1. The antibodies were either purchased from Standard Biotools or conjugated in-house using Maxpar® X8 Antibody Labeling Kits (Standard Biotools) according to the manufacturer's instructions. After conjugation, antibodies were eluted in 200mL of antibody stabilizer PBS (Candor Biosciences, 131125) containing 0.05% sodium azide and stored at 4°C. All antibodies were previously validated by our group(1-3).

1.1.3 Mass cytometry antibody staining and data acquisition

Cryopreserved BMMC or PBMC samples were measured in six batches. Samples collected from AA^{PRE} and HDs were paired and measured across five batches. BMMC samples taken from AA^{POST} were measured in the sixth batch. A cryopreserved PBMC reference sample was included in batches one and six to account for technical variation. All samples were stained and measured as previously described(3, 4). In brief, cryopreserved BMMCs or PBMCs were thawed, washed, resuspended in 2mL of cell staining buffer (Standard Biotools, cat 201068) and incubated with 1mL of 1:500 Cell-ID™ Intercalator-^{103}Rh 500µM (Standard Biotools, cat 201103A) for 15 minutes at room temperature (RT) to label dead cells. Subsequently, cells were washed, incubated with 5µL of Fc block™ (BD Biosciences, cat 564219) and stained with the 39 metal-isotope tagged antibodies of the mass cytometry panel in a volume of 100µL at RT. After a 45-minute incubation, cells were washed and 1mL of 1:1000 125nM of Cell-ID™ Intercalator-Ir (Standard Biotools, cat 201192A) in fix and perm buffer (Standard Biotools, cat 201067) was added to label nucleated cells. Cells were then stored at 4°C for up to 48 hours. On the day of data acquisition, cells were washed twice in staining buffer and once in MilliQ water. Finally, pelleted cells were resuspended in MilliQ water containing 1:10 EQ Four Element Calibration Beads (Standard Biotools, cat 201078) at a concentration of 0.5-$0.75 \cdot 10^6$ cells/mL and acquired on the Helios™ mass cytometer (Standard Biotools). At least $0.59 \cdot 10^6$ (median $0.88 \cdot 10^6$) events were measured per sample. After measurement, all mass

cytometry data were normalized using the EQ bead signal and the reference EQ passport P13H2302.

1.1.4 Analysis of pre- and post-ATG samples

To compare the immune cell composition in bone marrow pre- and post-ATG, paired BMMC samples collected from AA^{PRE} (n=3) and AA^{POST} (n=3) were studied. First, marker distributions across all data files with single, live $CD45^{+}$ cells were aligned using CytoNorm(5) in R version 4.0.5 (R foundation for Statistical Computing) and the PBMC reference samples to correct for a batch effect observed in the data of all post-ATG samples (data not shown). Outputs were checked and confirmed in FlowJo. Next, $3.0 \cdot 10^{6}$ single, live $CD45^{+}$ cells were sample-tagged, hyperbolic ArcSinh transformed with a cofactor of 5 and imported in Cytosplore for dimensionality reduction. A four-level HSNE analysis was performed with default perplexity and iterations (30 and 1000, respectively). Three of 39 markers in the mass cytometry panel (HLA-DR, CD20 and CD8a) did not show clear separation between positive and negative populations and thus were not used in the HSNE clustering analysis. Major immune lineages were identified at the overview level of the HSNE analysis by clustering. $CD4^{+}$ T-cells were defined as $CD3^{+}CD7^{+}CD4^{+}$ cells, $CD8^{+}$ T-cells were defined as $CD3^{+}CD7^{+}CD8a/b^{+}$ cells, B-cells were defined as $CD7^{-}CD20/IgM^{+}$ cells, non-conventional T-cells (NCTs) were defined as $CD3^{+}CD7^{+}TCRgd^{+}$ cells, innate lymphoid cells (ILCs) including NK-cells were defined as $CD3^{-}CD7^{+}$ cells and HSPCs were defined as $Lin^{-}cKit^{+}$ cells. All remaining cell clusters were considered myeloid cells. Subsequently, each major immune lineage was selected for detailed analysis at the data level of the HSNE analysis using t-distributed stochastic neighbor embedding (tSNE) analyses with up to $0.5 \cdot 10^{6}$ landmarks. Clusters with distinct surface marker expression were identified within each major immune lineage by performing Gaussian mean shift (GMS) clustering at the data level in Cytosplore. Each cluster consisted of at least 1000 cells.

References

1. Li N, van Unen V, Hollt T, Thompson A, van Bergen J, Pezzotti N, et al. Mass cytometry reveals innate lymphoid cell differentiation pathways in the human fetal intestine. J Exp Med. 2018;215(5):1383-96.

2. van der Zwan A, van Unen V, Beyrend G, Laban S, van der Keur C, Kapsenberg HJM, et al. Visualizing Dynamic Changes at the Maternal-Fetal Interface Throughout Human Pregnancy by Mass Cytometry. Front Immunol. 2020;11:571300.

3. van Unen V, Li N, Molendijk I, Temurhan M, Hollt T, van der Meulen-de Jong AE, et al. Mass Cytometry of the Human Mucosal Immune System Identifies Tissue- and Disease-Associated Immune Subsets. Immunity. 2016;44(5):1227-39.

4. Bendall SC, Simonds EF, Qiu P, Amir el AD, Krutzik PO, Finck R, et al. Single-cell mass cytometry of differential immune and drug responses across a human hematopoietic continuum. Science. 2011;332(6030):687-96.

5. Van Gassen S, Gaudilliere B, Angst MS, Saeys Y, Aghaeepour N. CytoNorm: A Normalization Algorithm for Cytometry Data. Cytometry A. 2020;97(3):268-78.

2 Supplementary tables and figures

Supplementary Table 1. 39-marker mass cytometry panel.

Metal	Marker	Clone	Vendor	Dilution
89Y	CD45	HI30	Standard Biotools	1:100
Qdot800	CD14	TuK4	Invitrogen	1:1000
115In	CD15	W6D3	BioLegend	1:50
141Pr	CCR6	G034E3	Standard Biotools	1:100
142Nd	CD34	HIB19	BioLegend	1:100
143Nd	cKit (CD117)	104D2	Standard Biotools	1:100
144Nd	CD69	FN50	Standard Biotools	1:100
145Nd	CD4	RPA-T4	Standard Biotools	1:100
146Nd	CD8a	RPA-T8	Standard Biotools	1:200
147Sm	NKp44 (CD336)	253415	R&D systems	1:40
148Nd	CD16	3G8	Standard Biotools	1:100
149Sm	CD25	2A3	Standard Biotools	1:100
150Nd	IgM	MHM88	BioLegend	1:100
151Eu	CD123	6H6	Standard Biotools	1:100
152Sm	TCRgd	11F2	Standard Biotools	1:50
153Eu	CD7	CD7-6B7	Standard Biotools	1:100
154Sm	CD163	GHI/61	Standard Biotools	1:100
155Gd	CD103	Ber-ACT8	BioLegend	1:100
156Gd	CRTH2 (CD294)	BM16	BioLegend	1:100
158Gd	CD122	TU27	BioLegend	1:50
159Tb	CCR7	G043H7	Standard Biotools	1:100
160Gd	CD5	UCHT2	BioLegend	1:50
161Dy	KLRG1	REA261	MACS	1:50
162Dy	CD11c	Bu15	Standard Biotools	1:200
163Dy	CD20	2H7	BioLegend	1:200
164Dy	CD161	HP-3G10	Standard Biotools	1:100
165Ho	CD127	AO19D5	Standard Biotools	1:200
166Er	CD8b	SIDI8BEE	eBioscience	1:50
167Er	CD27	O323	Standard Biotools	1:100
168Er	HLA-DR	L243	BioLegend	1:300
169Tm	CD45RA	HI100	Standard Biotools	1:100
170Er	CD3	UCHT1	Standard Biotools	1:100
171Yb	CD28	CD28.2	BioLegend	1:100
172Yb	CD38	HIT2	Standard Biotools	1:200
173Yb	CD45RO	UCHL1	BioLegend	1:100
174Yb	NKp46 (CD335)	9E2	BioLegend	1:40
175Lu	PD-1 (CD279)	EH 12.2H7	Standard Biotools	1:100
176Yb	CD56	NCAM16.2	Standard Biotools	1:100
209Bi	CD11b	ICRF44	Standard Biotools	1:100

CCR indicates chemokine receptor; CD: cluster of differentiation; CRTH2: Chemoattractant Receptor-Homologous molecule expressed on T-Helper Type 2 cells; HLA: Human Leukocyte Antigen; Ig: immunoglobulin; KLRG: Killer Cell Lectin-like Receptor; NKp44: Natural Killer Cell P44-related Protein; TCR: T-cell receptor; NKp46: Natural Killer Cell P46-related Protein; PD: Programmed Cell Death Protein.

Figure S1. Mass cytometry gating strategy used to identify single, live $CD45^+$ cells in BM aspirates. $CD45^+$ cells were first gated using CD45 and a DNA marker (panel 1). Subsequently, beads (panel 2), dead cells (panel 3) and doublets and debris (panels 4-6) were eliminated and single, live CD45+ cells (panel 7) were exported for analysis. The gating strategy on a representative HD BM aspirate sample is shown.

Figure S2. Approach used to analyze each major immune lineage at the immune subset level. HSPCs and myeloid cells are shown as an example. $1.3 \cdot 10^6$ HSPCs and myeloid cells isolated from BM aspirates of 7 AA^{PRE} and 7 HDs were captured at the overview level of the five-level HSNE analysis (Figure 1) and visualized in more detail at levels two ($0.7 \cdot 10^5$ landmarks for $1.3 \cdot 10^6$ cells), three ($4.2 \cdot 10^5$ landmarks for $1.3 \cdot 10^6$ cells) and four ($2.4 \cdot 10^5$ landmarks for $1.3 \cdot 10^6$ cells) of the HSNE analysis. Subsequently, all HSPCs and myeloid cells were divided into three clusters of $\leq 0.5 \cdot 10^6$ cells and studied at the data level. Next, distinct HSPCs and myeloid cell clusters were classified by Gaussian mean shift (GMS) clustering in Cytosplore based on cell densities and differences in marker expression. Finally, marker expression profiles and frequencies of identified clusters were studied in R. Each cluster consisted of at least 1000 cells.

Figure S3. **AA^{PRE} and HDs samples visualized at the overview level of the five-level HSNE analysis.** Colors indicate major immune lineages (HSPCs, myeloid cells, B-cells, $CD4^+$ T-cells, $CD8^+$ T-cells, NCT-cells and ILCs), samples (7 AA^{PRE} and 7 HDs) or groups (AA^{PRE} in red and HDs in gray). $7.8 \cdot 10^3$ HSNE landmarks represented all $7.7 \cdot 10^6$ $CD45^+$ cells in the analysis. The size of each landmark reflects the number of cells represented by the landmark.

Figure S4. Overview of the myeloid cell, B-cell, $CD4^+$ T-cell and $CD8^+$ T-cell lineages. Colors indicate major immune lineages (HSPCs, myeloid cells, B-cells, $CD4^+$ T-cells, $CD8^+$ T-cells, NCT-cells and ILCs), groups (AA^{PRE} or HDs) or median ArcSinh-transformed marker expression values. A, C, F, I) Phenotypes of the cell clusters identified within the myeloid, B-cell, $CD4^+$ T-cell and $CD8^+$ T-cell compartments, respectively. Heatmaps present median ArcSinh-transformed marker expression values. The relationships of similarity between cell clusters are depicted by the dendrogram. B, D, G, J) Frequencies of the cell clusters identified within the myeloid cell, B-cell, $CD4^+$ T-cell and $CD8^+$ T-cell compartments, respectively. Frequencies are presented per group and are shown as a percentage of all cells within a lineage. E, H) $CD4^+$ T-cells and $CD8^+$ T-cells isolated from BM of 7 AA^{PRE} and 7 HDs, visualized at level four of a five-level HSNE analysis. HSNEs depict $3.4 \cdot 10^5$ landmarks for $1.9 \cdot 10^6$ $CD4^+$ T-cells and $3.9 \cdot 10^5$ landmarks for $2.1 \cdot 10^6$ $CD8^+$ T-cells. The size of each landmark reflects the number of cells represented by the landmark. Arrows in (E) indicate $CCR6^+$ CM and EM CD4+ T-cells. RORA: T-cells co-expressing CD45RA and CD45RO.

Figure S5. Disease specific immune cell network can be detected in PB. Frequencies of the five cell clusters within the identified disease-specific immune cell network, shown in BM and PB. Paired BM and PB samples from AA^{PRE} are connected by a dotted line. BM and PB samples were compared in an independent analysis to avoid an influence of PB cells on clustering of BM cells from AA^{PRE} and HDs. Therefore, small differences in cell percentages may be observed between the data presented here and in Figures 2, 3, 4 and 5. The paired BM and PB samples were compared in a five-level HSNE analysis with default perplexity and iterations (30 and 1000, respectively) and a total of $11.5 \cdot 10^6$ single, live $CD45^+$ cells (BM samples from AA^{PRE} and HDs, and PB samples from AA^{PRE} only). Three of 39 markers in the mass cytometry panel (HLA-DR, CD5 and KLRG1) were not used to generate the HSNE due to small variations in marker distribution between experiments. Major immune lineages were identified at the overview level of the HSNE analysis by clustering based on the phenotypes described in the methods. Subsequently, each major immune lineage was selected for detailed analysis at the data level using tSNE analyses with up to $0.5 \cdot 10^6$ landmarks. Immune cells that matched the phenotypes of the $CD16^+$ myeloid cells, $CCR6^{++}$ B-cells, $CCR6^+$ memory $CD4^+$ T-cells and $CD45RA^+CCR7^+CD38^+$ $CD8^+$ T-cells and $KLRG1^+$EMRA $CD8^+$ T-cells that were significantly elevated in AA^{PRE} were captured in Cytosplore. Subsequently, their total frequencies were compared between BM and PB for each individual patient.

Figure S1

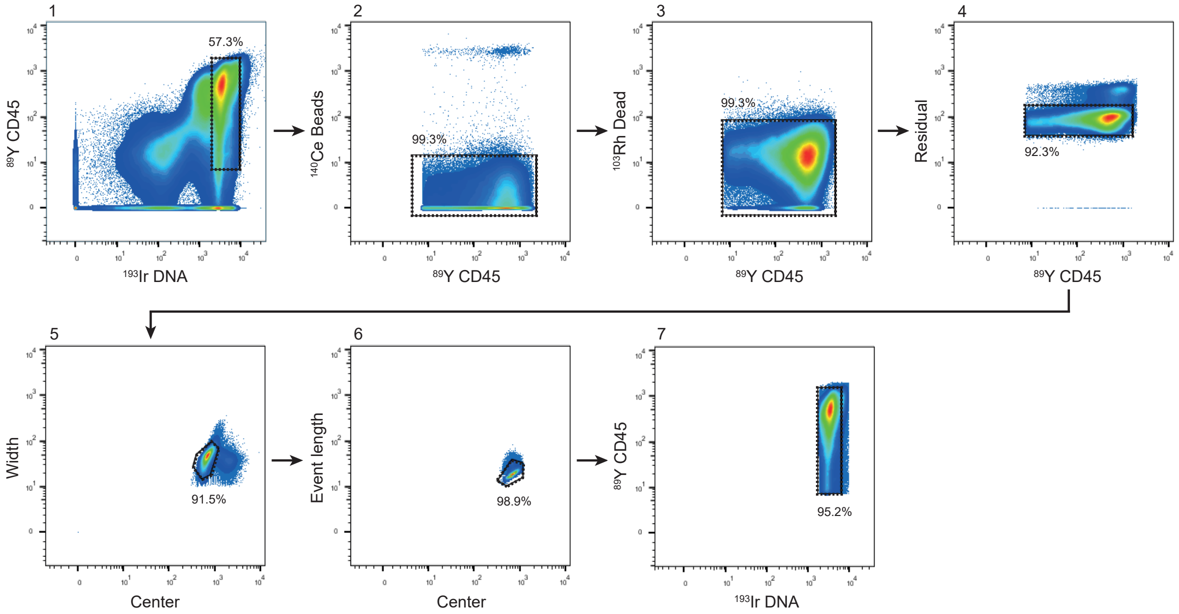

1
57.3%
89Y CD45
193Ir DNA
2
99.3%
140Ce Beads
89Y CD45
3
99.3%
103Rh Dead
89Y CD45
4
92.3%
Residual
89Y CD45
5
91.5%
Width
Center
6
98.9%
Event length
Center
7
95.2%
89Y CD45
193Ir DNA

Figure S2

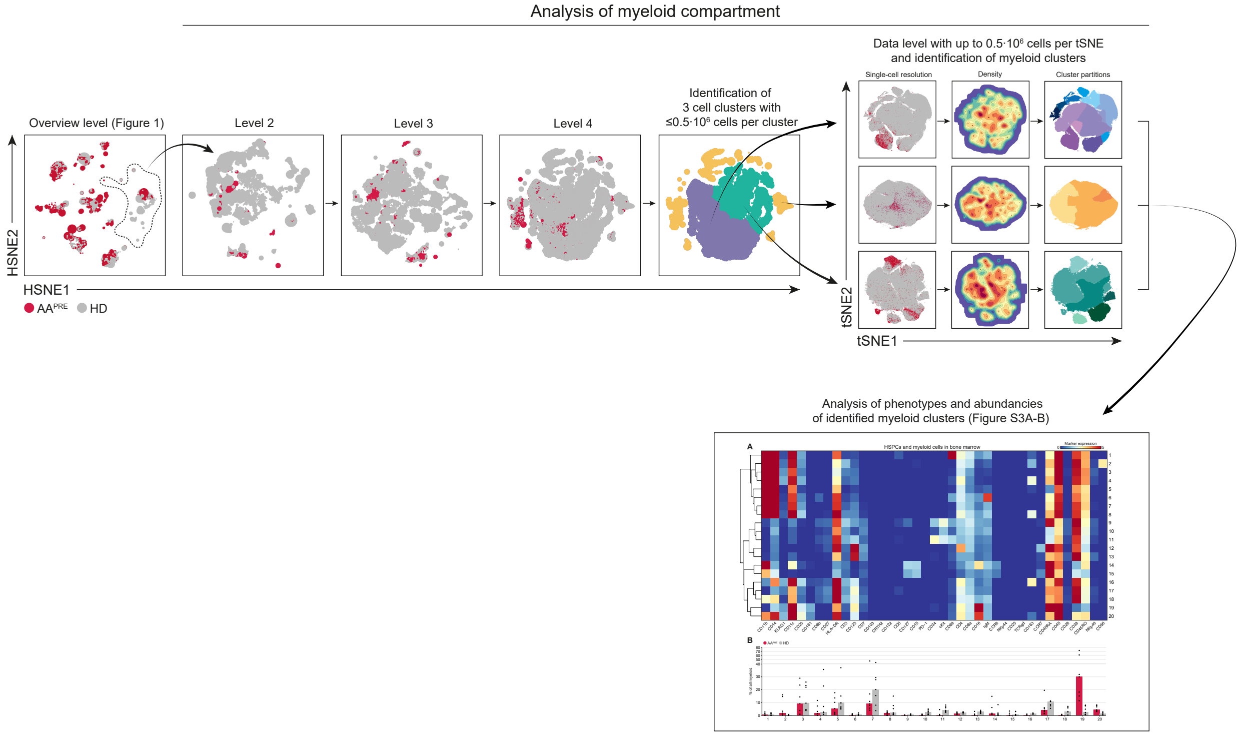
Analysis of myeloid compartment
Overview level (Figure 1)
Level 2
Level 3
Level 4
Identification of 3 cell clusters with ≤0.5·10⁶ cells per cluster
Data level with up to 0.5·10⁶ cells per tSNE and identification of myeloid clusters
Single-cell resolution
Density
Cluster partitions
HSNE2
HSNE1
AA PRE
HD
tSNE2
tSNE1
Analysis of phenotypes and abundancies of identified myeloid clusters (Figure S3A-B)
A
HSPCs and myeloid cells in bone marrow
B

Figure S3

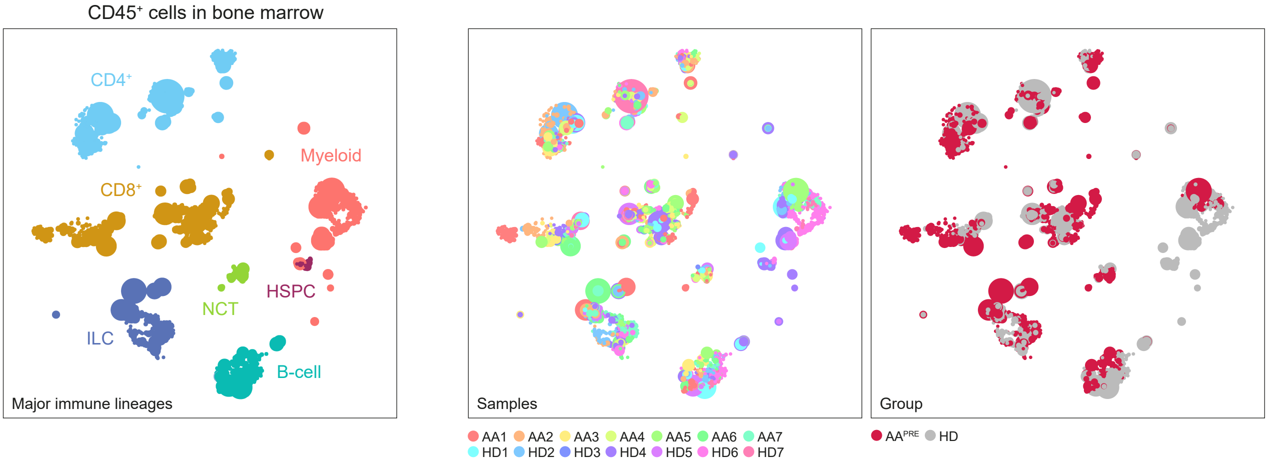
CD45⁺ cells in bone marrow
CD4⁺
CD8⁺
Myeloid
HSPC
NCT
ILC
B-cell
Major immune lineages
Samples
AA1 AA2 AA3 AA4 AA5 AA6 AA7
HD1 HD2 HD3 HD4 HD5 HD6 HD7
Group
AAPRE HD

Figure S4

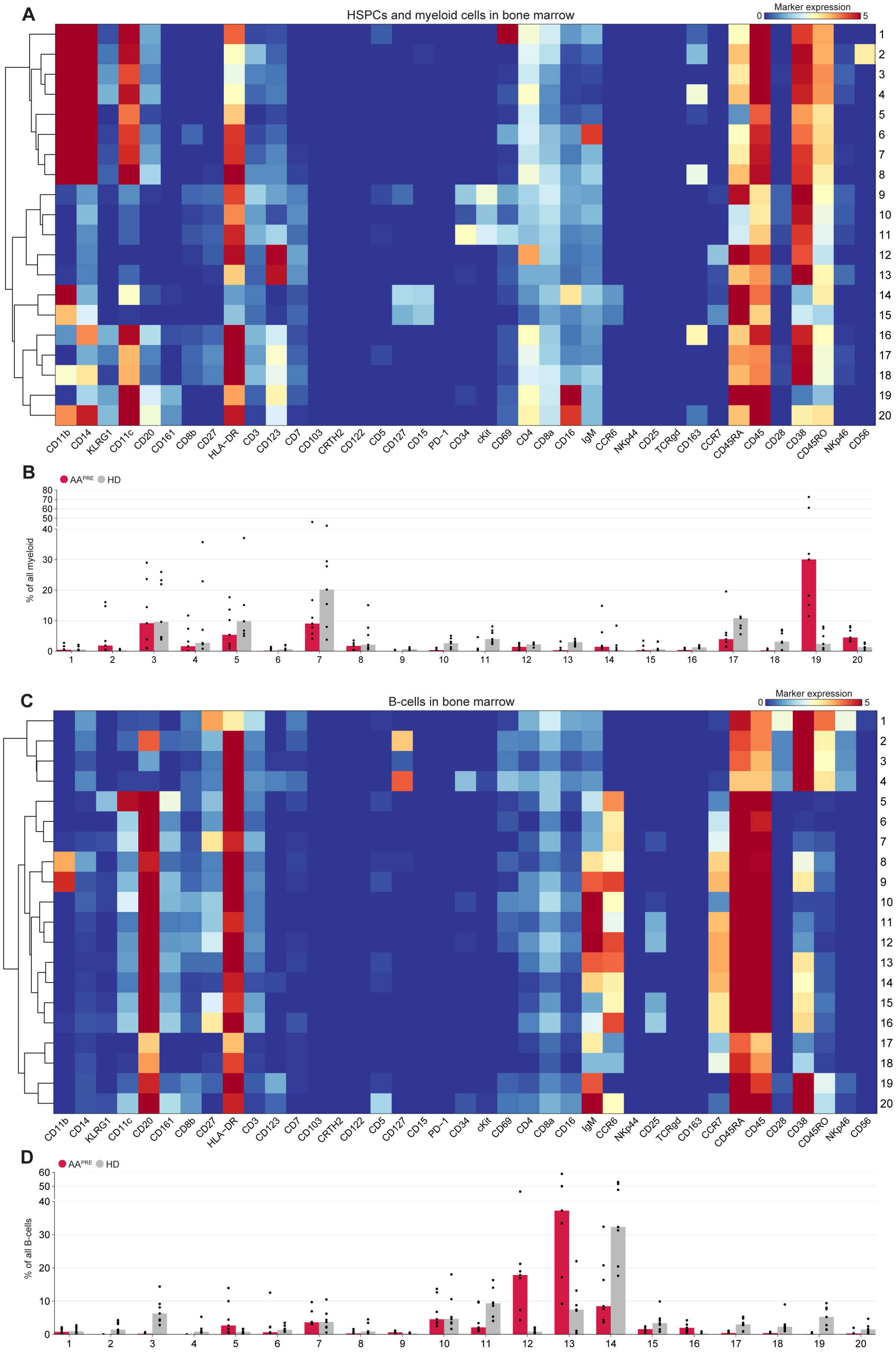

A
HSPCs and myeloid cells in bone marrow
Marker expression
0
5
CD11b CD14 KLRG1 CD11c CD20 CD161 CD8b CD27 HLA-DR CD3 CD123 CD7 CD103 CRTH2 CD122 CD5 CD127 CD15 PD-1 CD34 cKit CD69 CD4 CD8a CD16 IgM CCR6 NKp44 CD25 TCRgd CD163 CCR7 CD45RA CD45 CD28 CD38 CD45RO NKp46 CD56
B
AAPRE
HD
% of all myeloid
C
B-cells in bone marrow
Marker expression
D
AAPRE
HD
% of all B-cells

E

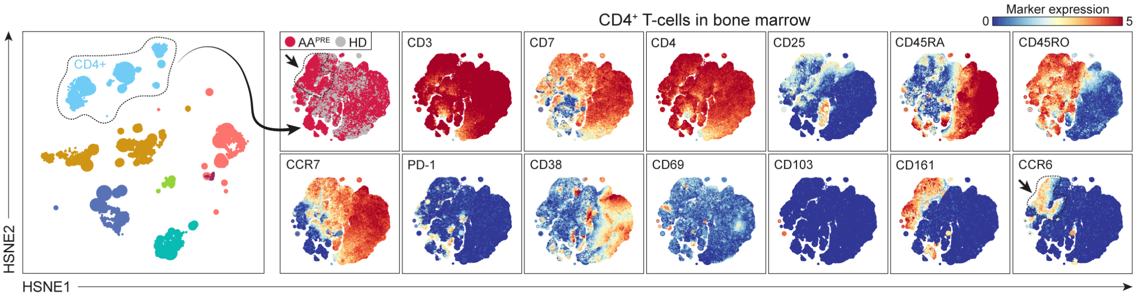
CD4⁺ T-cells in bone marrow
Marker expression
0
5
CD4+
AAPRE
HD
CD3
CD7
CD4
CD25
CD45RA
CD45RO
CCR7
PD-1
CD38
CD69
CD103
CD161
CCR6
HSNE2
HSNE1

F

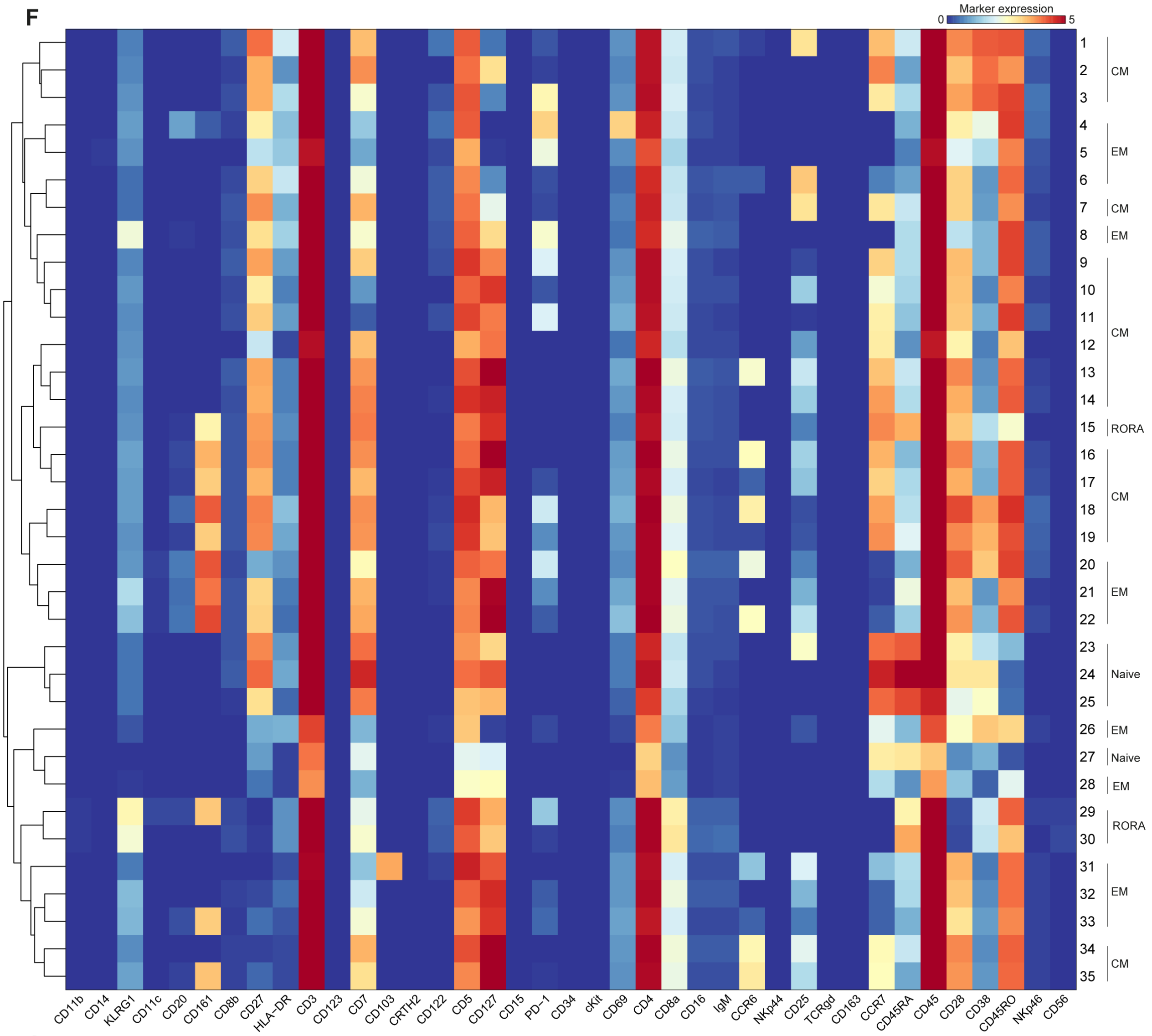
Marker expression
0
5
1
2
3
4
5
6
7
8
9
10
11
12
13
14
15
16
17
18
19
20
21
22
23
24
25
26
27
28
29
30
31
32
33
34
35
CM
EM
CM
EM
CM
RORA
CM
EM
Naive
EM
Naive
EM
RORA
EM
CM
CD11b
CD14
KLRG1
CD11c
CD20
CD161
CD8b
CD27
HLA-DR
CD3
CD123
CD7
CD103
CRTH2
CD122
CD5
CD127
CD15
PD-1
CD34
cKit
CD69
CD4
CD8a
CD16
IgM
CCR6
NKp44
CD25
TCRgd
CD163
CCR7
CD45RA
CD45
CD28
CD38
CD45RO
NKp46
CD56

G

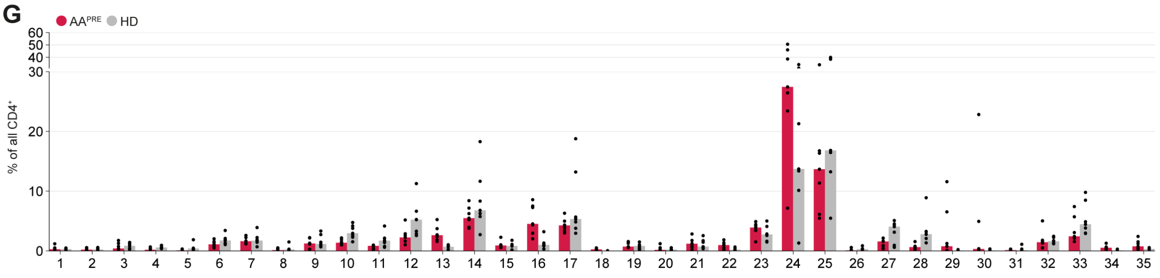
AAPRE
HD
% of all CD4⁺
60
50
40
30
20
10
0
1
2
3
4
5
6
7
8
9
10
11
12
13
14
15
16
17
18
19
20
21
22
23
24
25
26
27
28
29
30
31
32
33
34
35

H

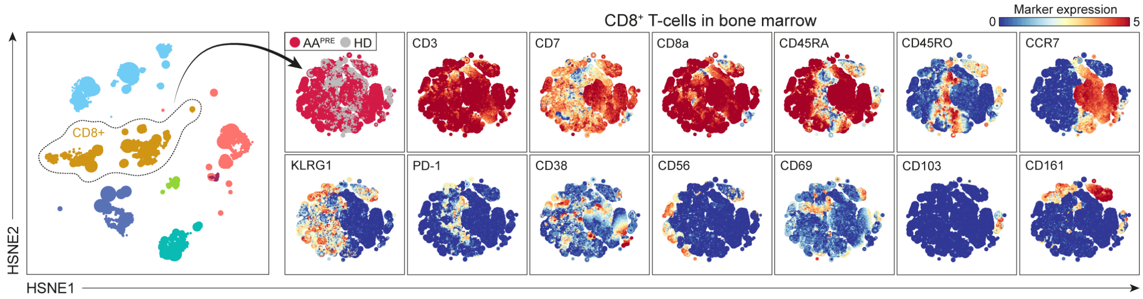
CD8+ T-cells in bone marrow
Marker expression
0
5
AAPRE
HD
CD8+
HSNE2
HSNE1
CD3
CD7
CD8a
CD45RA
CD45RO
CCR7
KLRG1
PD-1
CD38
CD56
CD69
CD103
CD161

I

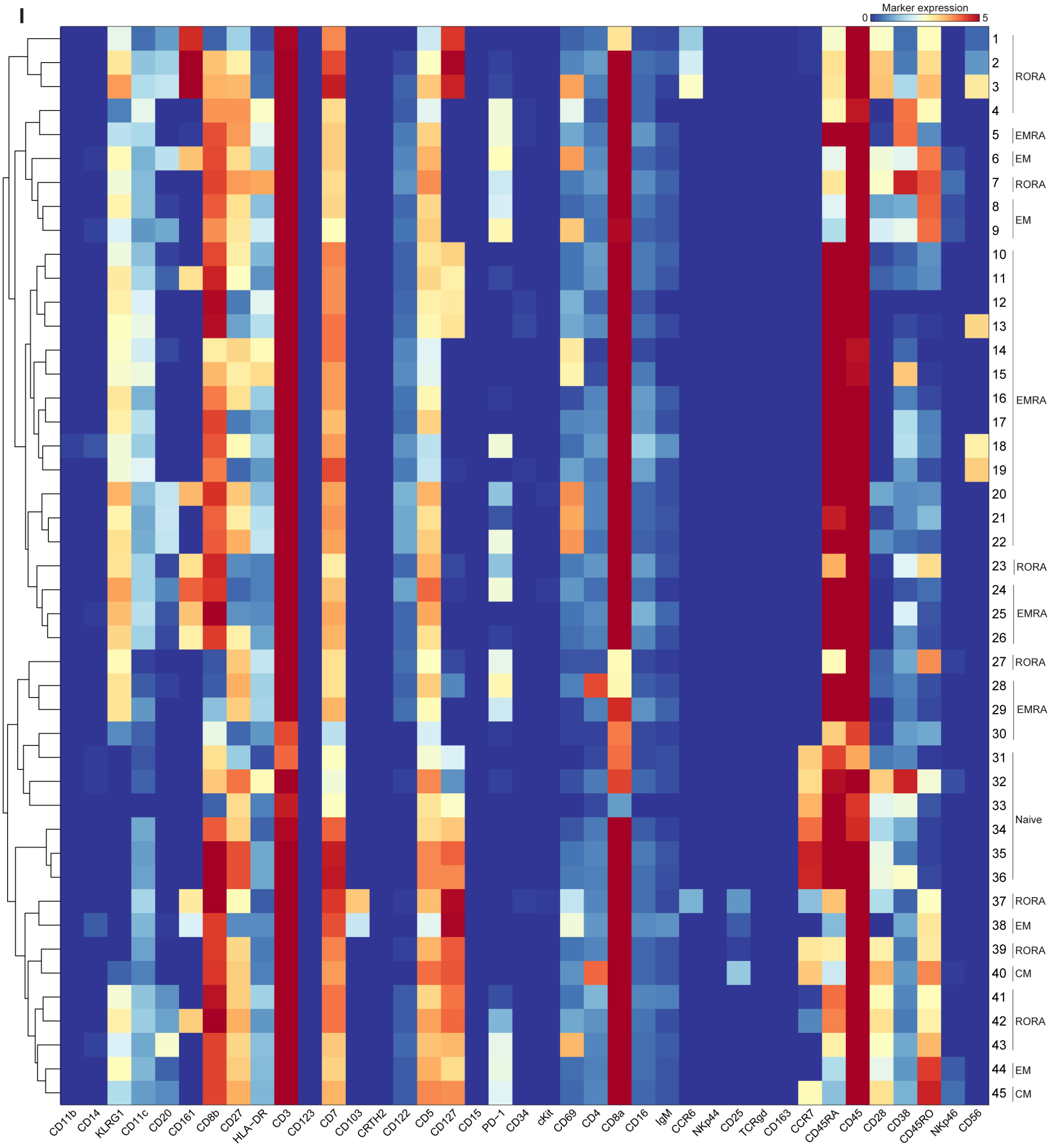
Marker expression
0
5
CD11b
CD14
KLRG1
CD11c
CD20
CD161
CD8b
CD27
HLA-DR
CD3
CD123
CD7
CD103
CRTH2
CD122
CD5
CD127
CD15
PD-1
CD34
cKit
CD69
CD4
CD8a
CD16
IgM
CCR6
NKp44
CD25
TCRgd
CD163
CCR7
CD45RA
CD45
CD28
CD38
CD45RO
NKp46
CD56
1
2
3
RORA
4
5 EMRA
6 EM
7 RORA
8
9
EM
10
11
12
13
14
15
16 EMRA
17
18
19
20
21
22
23 RORA
24
25 EMRA
26
27 RORA
28
29 EMRA
30
31
32
33
34 Naive
35
36
37 RORA
38 EM
39 RORA
40 CM
41
42 RORA
43
44 EM
45 CM

J

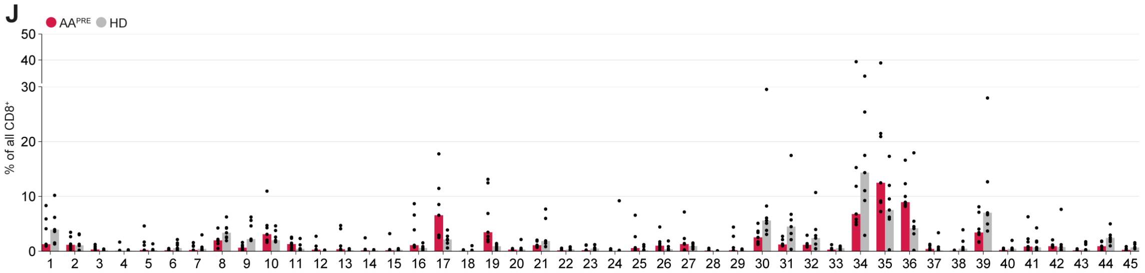
AA^PRE HD
% of all CD8+
0
10
20
30
40
50
1 2 3 4 5 6 7 8 9 10 11 12 13 14 15 16 17 18 19 20 21 22 23 24 25 26 27 28 29 30 31 32 33 34 35 36 37 38 39 40 41 42 43 44 45

Figure S5

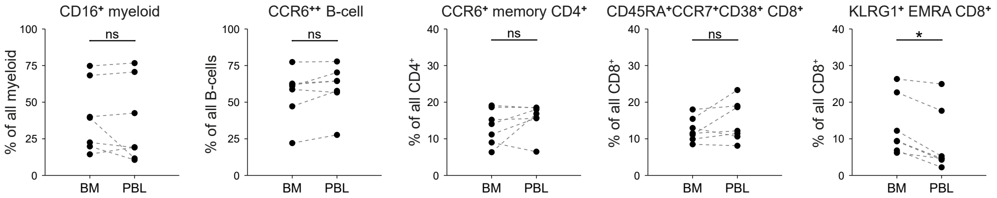

CD16⁺ myeloid
ns
% of all myeloid
100
75
50
25
0
BM
PBL
CCR6⁺⁺ B-cell
ns
% of all B-cells
CCR6⁺ memory CD4⁺
ns
% of all CD4⁺
40
30
20
10
CD45RA⁺CCR7⁺CD38⁺ CD8⁺
% of all CD8⁺
KLRG1⁺ EMRA CD8⁺
*
